# Central mechanisms of real and sham electroacupuncture in the treatment of chronic low back pain: study protocol for a randomized, placebo-controlled clinical trial

**DOI:** 10.1186/s13063-018-3044-2

**Published:** 2018-12-13

**Authors:** Jiang-Ti Kong, Brandon MacIsaac, Ruti Cogan, Amanda Ng, Christine Sze Wan Law, Joseph Helms, Rosa Schnyer, Nicholas Vasilis Karayannis, Ming-Chih Kao, Lu Tian, Beth D. Darnall, James J. Gross, Sean Mackey, Rachel Manber

**Affiliations:** 1 Division of Pain Medicine, Department of Anesthesiology, Perioperative and Pain Medicine, Stanford University School of Medicine, 1070 Arastradero Rd., Suite 200, Palo Alto, CA 94304 USA; 2 Helms Medical Institute, 2520 Milvia Street, Berkeley, CA 94704 USA; 3 The University of Texas at Austin, School of Nursing, Office, NUR 5.188, 1710 Red River Street, Austin, TX 78701 USA; 4 Department of Biomedical Data Science and Statistics, Stanford University, Stanford, CA 94305 USA; 5 Department of Psychology, Stanford University, Stanford, CA 94305 USA; 6 Department of Psychiatry and Behavioral Sciences, Sleep Center, 401 Quarry Rd Rm 3337, Stanford, CA 94305 USA

**Keywords:** Chronic, Low back pain, Electrical acupuncture, Electroacupuncture, Sham acupuncture, Quantitative sensory testing, Temporal summation, Expectation, Conditioned pain modulation, Pressure pain threshold, Catastrophizing, Self-efficacy, PROMIS

## Abstract

**Background:**

Chronic low back pain (CLBP) is the most common chronic pain condition and is often resistant to conventional treatments. Acupuncture is a popular alternative for treating CLBP but its mechanisms of action remain poorly understood. Evidence suggests that pain regulatory mechanisms (particularly the ascending and secondarily the descending pain modulatory pathways) and psychological mechanisms (e.g., expectations, pain catastrophizing and self-efficacy) may be involved in the pathogenesis of CLBP and its response to treatments. We will examine these mechanisms in the treatment of CLBP by electroacupuncture (EA).

**Methods:**

We present the aims and methods of a placebo-controlled, participant-blinded and assessor-blinded mechanistic study. Adult patients with CLBP will be randomized to receiving 16 sessions of real (active) or sham (placebo) EA over the course of 8 weeks. The primary pain regulatory measure for which the study was powered is temporal summation (TS), which approximates ascending pain facilitation. Conditioned pain modulation (CPM), representing a descending pain modulatory pathway, will be our secondary pain regulatory measure. The primary psychological measure is expectations of benefit, and the secondary psychological measures are pain catastrophizing and self-efficacy in managing pain. Main clinical outcomes are back pain bothersomeness on a 0–100 visual analog scale (primary), Roland Morris Disability Questionnaire (secondary), and relevant items from the National Institutes of Health (NIH) Patient-Reported Outcome Measures Information System (secondary). We hypothesize that compared to sham, real EA will lead to greater reduction in TS after 8 treatment sessions (4 weeks); and that reduction in TS (and secondarily, increase in CPM) after 8 treatment sessions will mediate reduction in back pain bothersomeness from baseline to week 10 (clinical response) to EA. We also hypothesize that the three psychological factors are moderators of clinical response. With 100 treatment completers, the study is designed to have 80% power to detect a medium-sized between-group effect (*d* = 0.5) on temporal summation.

**Discussion:**

To the best of our knowledge, this is the first appropriately powered, placebo-controlled clinical trial evaluating mechanisms of EA in the treatment of CLBP.

**Trial registration:**

ClinicalTrials.gov, NCT02503475. Registered on 15 July 15 2015. Retrospectively registered.

**Electronic supplementary material:**

The online version of this article (10.1186/s13063-018-3044-2) contains supplementary material, which is available to authorized users.

## Background

Chronic low back pain (CLBP) affects between 10 and 20% of adults [1], is the number one reason for missing work due to disability [2], and is the third most costly medical condition in the USA, after diabetes mellitus and ischemic heart disease [3]. The management of CLBP remains a significant challenge. Conventional, anatomically-based treatments such as surgical fusion and disc replacement often result in minimal or short-lived improvement [4]. Past research has determined that many patients with CLBP may have abnormalities in pain processing [5–7].

Electroacupuncture (EA) offers a promising alternative to conventional approaches in treating CLBP (i.e., drugs and surgery) and needs additional investigation. The majority of studies on acupuncture for CLBP have used manual acupuncture with equivocal results [8]. For example, both Haake (Germany) [9] and Cherkin (USA) [10] found that acupuncture led to greater improvement in pain and function compared with usual care (medical visits, physical therapy, and oral medications). However, neither study demonstrated a statistically significant difference between real and sham acupuncture. Pre-clinical and animal studies have suggested that EA might have stronger analgesic effects compared with manual acupuncture [11]. However, there is a relative paucity of studies on the effectiveness and even fewer studies on the mechanisms of EA in treating chronic pain conditions, particularly CLBP. Our study aims to address this gap by examining the differential mechanisms of real vs sham EA in the context of what is to the best of our knowledge, the largest randomized controlled, blinded study comparing the two treatments.

Central pain processing: the central processing of pain (i.e., nociceptive signals) has an ascending (peripheral to central) and a descending (central to peripheral) modulatory pathway [12–16]. There are both facilitatory and inhibitory inputs for each pathway. The final pain perception is the result of the integration of these complex bidirectional inputs from both the ascending and the descending pathways. Dysregulation in these pathways has been associated with CLBP [5, 6, 17]. Normalization of central pain processing [18–20] has been the mechanism purported to explain the success of several treatments for CLBP, including antidepressants, anticonvulsants, and cognitive behavioral therapy [21–23].

It is difficult to measure central pain processing directly in humans. Several experimental, non-invasive techniques have been used to infer central pain processing. One key technique is quantitative sensory testing (QST). It consists of administering standardized sensory stimuli to participants and measuring their ratings of the intensity of these stimuli and pain thresholds [24, 25]. Temporal summation (TS), to be used in this study as the primary mechanism outcome, is a specific QST measure that estimates ascending pain facilitation at the spinal cord level. Specifically, TS refers to increased pain perception to the application of a rapid sequence of identical noxious stimuli [26]. The increase in pain perception is considered a behavioral correlate of windup, a physiological process where wide-dynamic range (WDR) neurons in the spinal dorsal horn augment incoming rapid pain signals. Windup occurs when there is insufficient time for the stimulated WDR neurons to return to their baseline state before the next stimulus is applied [27, 28]. Windup is a key step in the ascending facilitation of pain transmission.

Secondarily, we will also assess conditioned pain modulation (CPM). CPM is a QST measure of the descending inhibitory central pain pathway. The CPM quantifies the extent to which the application of a second noxious stimulus reduces the perception of pain from a prior noxious stimulus [29]. In that way, CPM is considered the behavioral correlate of diffuse noxious inhibitory control, where a single, heterotopic noxious stimulus leads to brain-stem-mediated diffuse inhibition of subsequent incoming nociceptive transmission from elsewhere in the body [30]. Diffuse noxious inhibitory control is distinct from distraction [31].

Preliminary studies in animals and healthy human subjects suggest involvement of the central pain modulatory pathway in acupuncture analgesia [32]. For example, studies by Han [33] and Pomeranz [34] independently revealed that acupuncture, particularly electroacupuncture (EA), produces pain relief by augmenting descending pain inhibition via central opioidergic receptors at the periaqueductal gray and spinal dorsal horn. Similarly, Zheng et al. showed that acupuncture reduces ascending pain facilitation, as evidenced by decreases in TS from electrical pain induced in healthy volunteers [35]. They further showed that EA produces greater reductions in TS than manual acupuncture. Most of these studies were conducted on animals or healthy volunteers [36]. In this innovative study, we propose to investigate central mechanisms of acupuncture in a clinical population suffering from CLBP.

Expectations: several clinical studies have found that pretreatment expectation of benefits from treatment is a powerful predictor of pain reduction in response to treatments [37, 38]. Sherman et al. found that expectation following initial treatment might be more relevant to eventual outcome than pretreatment expectations [39]. In the present study, we will assess expectations both after (primary) and before (secondary) the first treatment session and examine both post first-session (primary) and pre first-session (secondary) expectations as moderators of clinical outcomes.

Additional (secondary) psychological factors: some data suggest that psychological and cognitive attributes, such as perceived self-efficacy in managing pain and pain catastrophizing, may also moderate clinical outcomes in response to pain treatment in general [40, 41] and acupuncture in particular [42]. Therefore, we will assess these two constructs and examine them as moderators of clinical outcomes.

### Specific aims and hypotheses

#### Aim 1

Aim 1 is to characterize the effect of TS (primary pain regulatory measure) as a mediator of reduction in back pain bothersomeness in response to treatment (primary clinical outcome). We hypothesize that:

(a) Compared to sham, real EA will lead to greater reduction in TS from baseline (week 0) to the end of week 4 (i.e., after 8 biweekly treatment sessions) (aim 1a)

(b) The change in TS from baseline to week 4 will mediate reduction in back pain bothersomess over the course of treatment (from baseline to post treatment (around week 10)) (aim 1b)

#### Aim 2

Aim 2 is to assess expectation of benefits (primary psychological measure) as a moderator of reduction in back pain bothersomeness in response to treatment (primary clinical outcome). We hypothesize that participants’ expectations of benefits will predict reduction in back pain bothersomeness scores during the treatment period (week 0 to week 10).

#### Aim 3 (exploratory)

Aim 3 is to assess aims 1 and 2 using secondary mediator and moderator variables of reduction in back pain bothersomeness from week 0 to week 10 (primary outcome) and the reduction in secondary clinical outcome measures over the same time frame. Hypotheses to be tested are identical to the hypotheses for aims 1 and 2, replacing the primary mediator with secondary mediators and primary moderators with secondary moderator variables.

##### Clinical impact

This clinical trial aims to delineate pain regulatory and psychological mechanisms of real (active) versus sham (placebo) EA in treating CLBP. The primary mechanisms investigated in this study are ascending pain facilitation estimated by TS, a specific measure from quantitative sensory testing, and expectations of benefit from the intervention, a psychological measure. The primary clinical outcome in this mechanistic study is back pain bothersomeness. While we will provide results on comparative efficacy of the real and sham EA in treating CLBP, our primary focus is on mechanisms and predictors of the clinical outcomes.

The results of this mechanistic study could have future clinical care implications because they will inform on the potential contribution of pain regulatory processes (mediator) and expectation of benefit (moderator) to clinical outcome. Identification of mediators of clinical response could help guide selection of patients who are most likely to respond to EA. For example, those with deficits in a given mediator variable, such as those with temporal summation deficit may potentially have better response to acupuncture. Identification of moderators will help guide decisions about targets to enhance clinical outcome. For example, we can potentially augment clinical outcome from acupuncture by an intervention to enhance expectation of benefits.

## Methods

### Trial design

In this randomized, placebo-controlled clinical trial each participant will have an equal chance (50%) of being randomized to receive real or sham EA. Our goal is to examine the pain regulatory mechanisms of real (active) and sham (placebo) EA in treating CLBP and evaluate the role of expectations of benefit and other psychological variables in moderating clinical response.

### Explanation for choice of comparators

We will include a parallel, placebo control in the form of sham EA because in order to assess the specific effects of acupuncture, we need to control for the nonspecific effects of acupuncture [43, 44]. We are interested in elucidating both the nonspecific (delivered by sham and real EA) and the specific (delivered by real EA) elements of acupuncture analgesia.

### Study setting

This is a single-site study. Study screening, enrollment, and procedures will take place at the Stanford Center for Back Pain Laboratory. Acupuncture treatment administration will take place at community acupuncture clinics in the larger San Francisco Bay Area.

### Participants

The Inclusion criteria are:

1. Men or women between the ages of 21 and 65 years.
2. Fluent in English.
3. Present with CLBP as their primary pain complaint (chronic low back pain is defined in the context of this study as having persisted at least 3 months and having resulted in pain on at least half the days in the past 6 months. We used the NIH Research Standards for Back Pain as a guide [45].
4. Average back pain intensity reported at the time of the screening visit greater than or equal to 4 out of 10 during the preceding month.

The exclusion criteria are:

1. Previous acupuncture treatment for any reason in the past 5 years. Participants with recent acupuncture exposure are more likely to recognize real as compared to sham EA and become unblinded, thereby increasing bias in the outcome. We therefore elected to exclude those with recent acupuncture experience to help minimize unblinding and reduce bias.
2. Any radicular symptoms; any identified spinal nerve root or spinal cord disease contributing to back pain; co-morbid pain syndrome (secondary pain complaint not exclusionary if less severe).
3. Ongoing legal or disability claim, worker’s compensation (permanent and stationary disability not exclusionary)
4. Currently pregnant or planning to become pregnant.
5. Medical conditions that would interfere with study procedures (e.g., heart disease or pacemaker, active infection, current cancer diagnosis; bleeding disorders).
6. Psychiatric disorders, indicated by the Mini International Neuropsychiatric Interview (MINI) (version 7.0, including
  ○ Major depressive episode: current (2 weeks)
  ○ Suicidality: current (past month)
  ○ Manic episode: current
  ○ Hypomanic episode: current
  ○ Post-traumatic stress disorder (PTSD): current (past month)
  ○ Alcohol use disorder: past 12 months
  ○ Substance use disorder: past 12 months
  ○ Psychotic disorders: lifetime
  ○ Mood disorder with psychotic features: current
  ○ Anorexia nervosa: current (past 3 months)
  ○ Bulimia nervosa: current (past 3 months)
  ○ Antisocial personality disorder: lifetime
7. Medications: starting new medical treatment or medication for pain 2 months prior to initiation of study procedures (participants who start such treatment after enrollment will be discontinued); opioids (≥ 60 mg morphine equivalent units/day), benzodiazepines, beta-blockers, some antipsychotics, diabetic medications, other medications that may interfere with study procedures, determined at the discretion of the study team; (selective serotonin reuptake inhibitors, anticonvulsants, and thyroid medications are not exclusionary, but tricyclic antidepressants and serotonin and norepinephrine reuptake inhibitors will be excluded); blood thinners (e.g., Coumadin, Plavix), at the discretion of the study team.

### Interventions

Table 1 contains a summary of the details of our interventions in the format according to the revised Standards for reporting interventions in clinical trials of acupuncture (STRICTA). We will use a standardized approach to real EA (active intervention), both in terms of point selection and level of stimulation. A total of 20 needles will be used in each session. The majority of local (inner bladder meridian line in the lumbosacral area) and distal (KI-3 and 7) points will be stimulated electrically but a few points will be stimulated manually. We built in some guided flexibility in point selection, allowing the addition of up to 10 more needles to address cases where the participant reports hip/buttock pain, and/or if the participant does not experience at least 30% pain reduction after the first four sessions. Each participant cannot miss more than 3 of the 16 acupuncture sessions, to be considered as having completed treatment.

**Table 1:** Details of treatment parameters summarized per STRICTA (version 2010) [61]

Item	Detail
1. Acupuncture rationale (explanations and examples)	1a) Style of acupuncture: - French energetics, channel theories from Chinese medicine, and neuro-anatomical (percutaneous electrical nerve stimulation (PENS)) [62]
	1b) Reasoning for treatment provided: - Point selections are based on the book by Dr J Helms, publications, and expert consensus
	1c) Extent to which treatment is varied: - We allow flexibility to include hip points if the participant reports hip/buttock pain; we also allow up to 3 additional points to be added if participants do not report at least 30% pain reduction after 4 sessions.
2. Details of needling (explanations and examples)	2a) Number of needle insertions per subject per session: - 20 (up to 30 if hip pain is present and if there is no response by the fifth session
	2b) Names (or location if no standard name) of points used: - Obligatory: GV-20, 4, BL-23, 25, 26, 28, KI-3, 7, HR-3, SI-3, BL-40 - Optional: GB-30 if there is hip/buttock pain (laterality depends on location of that pain); BL-10, 60, KI-10 if there is no response by the fifth session
	2c) Depth of insertion: - 0.5 (GV-20) to 6 cm (GB-30), depending on the location and patient size (most fall between 1 and 2 cm)
	2d) Response sought: - Deqi
	2e) Needle stimulation: - After Deqi is achieved at each needle, the following needles will also be electrically stimulated for 20–25 min: BL-23, 25, 26, 28, KI-3, 7. Then, if hip/buttock pain is present, posterior superior iliac spine (PSIS) and GB-30 - All e-stims will be bipolar, DC, at 2 Hz, and will be titrated to cause gentle muscle twitch, or to the amount that the patient can comfortably tolerate for 20 min if twitch is not obtainable
	2f) Needle retention time: 30 min
	2 g) Needle type (diameter, length, and manufacturer or material): - Auricular: Seirin J15 (0.12 × 15 mm) - Gluteal (GB-30): Huaxia (0.3 × 75 mm) - All other body needles: DBC Spring-10 (0.2 × 40 mm)
3. Treatment regimen (explanations and examples)	3a) Number of treatment sessions: 16 sessions over 8 weeks
	3b) Frequency and duration of treatment sessions: - First session up to 90 min, including the initial assessment - All subsequent sessions will be 45 min long
4. Other components of treatment (explanations and examples)	4a) Details of other interventions administered to the acupuncture - Heat lamp to low back - Auricular acupuncture to Shenmen, point zero, thalamus, and lumbar spine points
	4b) Setting and context of treatment - Participants will be informed that they may receive either real or sham EA and that both have been shown to be helpful in relieving pain
5. Practitioner background (explanations and examples)	5) Description of participating acupuncturists: 1. Licensed and ensured to administer acupuncture in the State of California 2. At least 3 years of acupuncture experience prior to treating study participants 3. Attendance at a full-day training session at the Stanford Center for Back Pain, and half-day on-site training at their own facility by the protocol co-director (JK) 4. Adequate ability to follow both the real and placebo treatment administration protocols, as assessed by the protocol co-director (JK) 5. Adequate facility to provide acupuncture treatment, approved by the protocol co-director (JK)
6. Control or comparator interventions (explanations and examples)	6a. Rationale for the control or comparator: - To minimize specific and physiologic effects, non-penetrating, Streitberger needles will be used at non-acupuncture, off-meridian points [46] - Sham electrical stimulation will be delivered via real machines with broken wires, based on a validated protocol from Wayne et al. [48] - Sham auricular points will be treated by placing a small piece of tape at the top and bottom apex of the ear - Heap lamp will be placed over the calf for approximately 20–25 min
	6b. Precise description of the control or comparator: - Point location: S1--in the middle of deltoid, 3 Cun below the lateral edge of acromion; S2--5 Cun lateral to the T-12 spinous process; S3--5 Cun lateral to L-5 spinous process; S4--midpoint on the line connecting BL-57 and GB-34 on the posterior calf - Point stimulation: minimal palpation to locate point, no Deqi. All points (S1-S4) receive sham electrical stimulation, which is performed after the acupuncturist informs the participant that he/she may or may not feel the paresthesia, because sometimes therapeutic electrical treatments are below the sensory detection threshold - The duration of needle retention and sham stimulation will be identical to that of the real treatment - Treatment regimen: same frequency and total number of treatment sessions as the real treatment in item #3, above

The sham EA (i.e., placebo intervention) has the same duration and treatment frequency as the real EA. In order to minimize specific physiological effects from the placebo EA, non-penetrating Streitberger needles will be used [46], placed at non-meridian points (away from the center of the back), which involves no electrical stimulation. The choice for placement of the needles was informed by previous research showing that even non-penetrating touch at the location of pain could lead to pain relief due to the activation of specific, non-painful, tactile c-fibers [33, 47]. We also use sham electrical stimulation in the placebo control, where the needles are connected to functioning electrical stimulators via broken wires. This particular technique was successfully used by Wane et al [48].

Initiation of new non-study pain interventions (including surgeries, pain blocks, medications, physical therapy, or chiropractic regimens) will not be allowed during the intervention period. Participants who initiate a new intervention for pain will not be included in the final analysis. To ensure patient safety, study treatments will be discontinued in the unlikely event that the participant experiences persistent, serious side effects (e.g., nerve damage). Serious side effects are rare [49] and have not occurred so far.

Fidelity: the research team will monitor acupuncturists’ treatment fidelity. A review of a random 10–15% of the sessions for each acupuncturist will be performed quarterly. We will review both the structured case report forms completed by the acupuncturists after each session and audio recordings of the sessions. To minimize the potential for drift from the treatment protocol and optimize treatment fidelity, there will be quarterly meetings of the acupuncturists and the first author, where corrective feedback of protocol deviations, if detected, will be discussed.

### Measures

#### Overview

The primary mediator is TS. The primary moderator is participants’ expectations of treatment benefits after the first treatment session. The primary clinical outcome is self-reported back pain bothersomeness. The secondary QST mediator to be examined is CPM. Secondary psychological moderators include pretreatment expectation of benefits, pain catastrophizing and self-efficacy in managing pain. Secondary clinical outcomes include the Roland Morris Disability Index, and multiple domains from the Patient Reported Outcome Measures Information System (PROMIS) item bank assessing physical and emotional functioning, fatigue and sleep. All of these assessments except for back pain bothersomeness, which is measured weekly, occur at baseline, halfway through treatment (between the 8th and 9th treatments, around the end of week 4), and post treatment (within 2 weeks after the final treatment, around week 10).

We will also collect long-term follow-up data, including back pain bothersomeness and the Roland Morris Disability Questionnaire. These will be collected at 1, 3, 6, 9, and 12 months after the end of treatment. Although not a primary aim, we collect these measures to characterize the effect of the mechanisms of interest in mediating long-term clinical outcomes.

#### Primary measures

Temporal summation (TS) - ascending pain facilitatory mechanism (primary mediator): we will use an individualized thermal TS protocol, measured on the back and the hand of each participant [50]. Through a unique participant training process and steps that aim to optimize TS by adjusting the stimulus and baseline temperatures, our method minimizes floor and ceiling effects due to variability in heat sensitivity and enables the capture of TS in a broad range of individuals [51]. This protocol delivers a series of 10 individually adjusted, supra-threshold heat pulses (0.5 s duration, given every 2 s) to the participant’s palm. TS is estimated as the difference between the rating of the second pain from the first pulse from that of the most painful pulse. We will measure TS at baseline (pretreatment measurement), during the treatment period (between the 8th and 9th treatment session), and post treatment.

Expectation of benefit - psychological mechanism (primary moderator): the Stanford Expectation of Treatment Scale (SETS) is a validated measure that assesses both positive and negative expectancies. It has a total of 6 questions (3 assessing positive and 3 negative expectations) and all questions are answered by a 7-point Likert scale scored from 1 to 7. The Cronbach’s alpha was 0.81–0.88 for positive expectancies and 0.81–0.86 for negative expectancies measured by the SETS. Participants will complete SETS both before and after the first treatment session.

Self-reported back pain bothersomeness - primary clinical outcome: participants will rate weekly back pain bothersomeness using a visual analog (VAS) 0–100 scale, where 0 is no pain and 100 is the worst pain imaginable. The back pain bothersomeness scale is specific to back pain and has been successfully used by researchers in other clinical trials [10, 52]. This measure will be collected weekly from baseline (week 0) to the post-treatment visit (around week 10).

#### Secondary measures

Conditioned pain modulation - descending pain modulation mechanism (secondary mediator): our group has optimized and published a method to quantify CPM [51]. Briefly, we measure the change in the pain rating on application of a 30-s thermal stimulus calibrated to approximately 60/100 pain intensity of the participant, applied to his/her non-dominant hand, before and during the last 30 s of a 2-min submersion of the contralateral foot in a 10 °C water bath. The magnitude of CPM is estimated as the difference in the pain rating of the thermal stimulus before and during the cold submersion. Our previous study demonstrated that CPM measured this way is robust against cognitive influences such as reappraisal or imagery [53].

Secondary psychological moderators: we will used validated questionnaires including pain catastrophizing scales [54] and self-efficacy in pain management [55].

Secondary clinical outcome measures: these will include the Roland Morris Disability Questionnaire, which assesses physical function specific to patients with back pain, and the following PROMIS measures: pain interference, physical function, social isolation, depression, anxiety, fatigue, sleep disturbance, and sleep-related Impairment. The secondary psychological moderators and clinical outcomes will be collected in a manner identical to the QST measures at baseline, at the mid-point during treatment, and post treatment.

### Timeline for participants

**Fig. 1:**
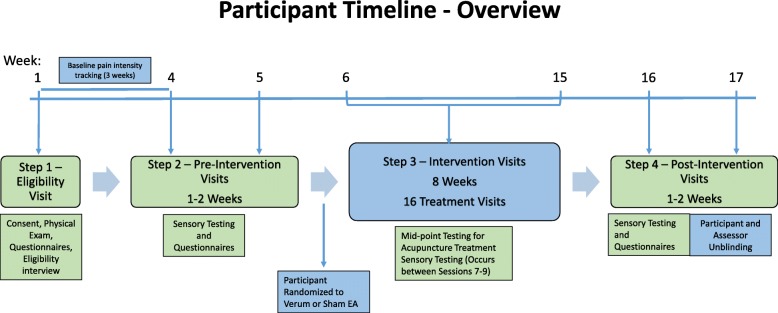
Participant timeline. EA, electroacupuncture. Participants are randomized to verum (real) or sham acupuncture

**Fig. 2:**
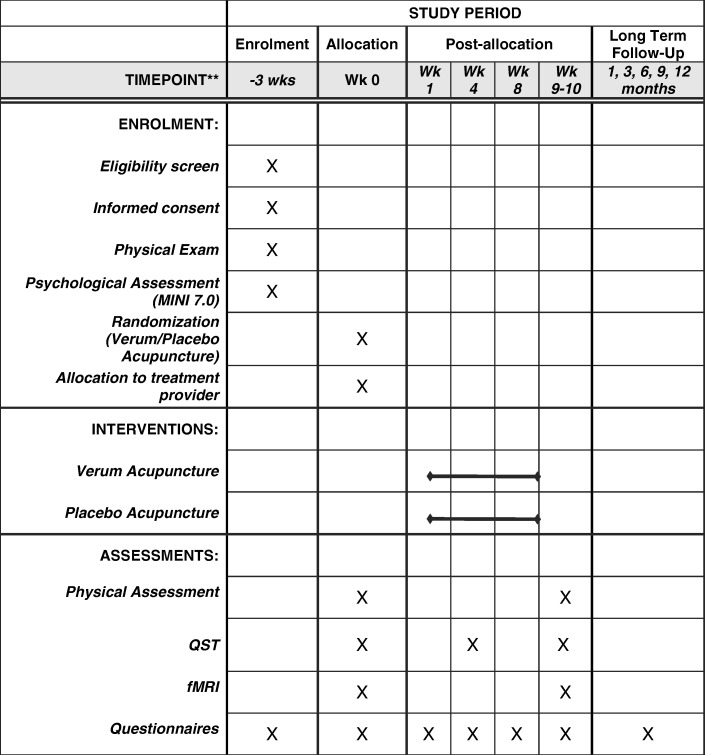
Standard protocol items: recommendation for interventional trials (SPIRIT) figure (required for study protocols). Wk, week; MINI, Mini International Neuropsychiatric Interview; QST, quantitative sensory testing; fMRI, functional magnetic resonance imaging

Figure 1 represents the participant’s timeline. Outlined in Fig. 2 (Additional file 1) is the study schedule with additional details per the SPIRIT CHECKLIST.

#### Sample size

We computed the sample size based on detecting differential changes in TS, the primary mediator. A small pilot study conducted in our laboratory suggested an average change of 10–25 points on the 100-point VAS with a standard deviation of 12 in TS following acupuncture. Prior studies suggest no treatment effect in controls receiving sham acupuncture [35]. However, given the imprecision of the pilot study estimates of effect sizes, we have elected to power the study to detect a medium-sized effect (*d* = 0.56) with 50 subjects in each arm, using the two-tailed test with alpha = 0.05 and beta = 0.8. If the point estimates from the pilot prove to be correct, this project will be very well-powered (98% power) to detect a large effect (*d* = 0.833). To account for attrition of about 20%, we plan to enroll about 120 study participants.

#### Recruitment

We will recruit participants via both online and physical venues. The online venues include social networks such as Facebook, advertising targets such as Craigslist, and research platforms such as Researchmatch.org. We also reach out to potential participants using newspaper and radio ads and flyers and brochures placed in physicians’ offices and community locations throughout the Bay Area. All interested individuals are directed to a secure, online REDCAP™ survey that functions as an initial screen for potential study participation.

#### Screening and consent

Initial eligibility will be assessed via the online REDCAP™ survey. Further eligibility will be assessed via a telephone screen based on the inclusion/exclusion criteria. Individuals who meet preliminary criteria will be scheduled for their screening visit. At the beginning of the screening visit, designated and trained research staff will review the Institutional Review Board (IRB)-approved consent and Health Insurance Portability and Accountability Act (HIPPA) forms with the participant in a private area and obtain informed consent. Research staff will allow ample time for the participant to ask questions so that the participant fully understands all the interventions and assessments associated with this study, and the benefits and risks of his/her participation. At the end of the visit, each participant will receive a signed and dated copy of their consent form and HIPPA documents.

#### Randomization, allocation concealment and blinding

We will randomize eligible participants to either the real or the sham intervention arm using a biased coin algorithm [56], which is an adaptive randomization process that aims to equalize the baseline pain level between the two groups. Given the influence of baseline pain level on clinical outcomes of pain studies [57], and our limited sample size, we decided to focus on equalizing levels of baseline pain severity, and not to equalize other factors, such as gender and age, which had less impact on the final outcome in past research [58, 59]. Each participant will be classified as having more or less extreme baseline pain (using 7/10 as a pain severity cutoff score). The algorithm will shift the randomization from a 50/50% chance of sending a participant with extreme pain into either treatment arm to having a 40/60% chance of assigning the participant with extreme pain into the arm that has more participants with extreme pain.

Participants will be randomized by the research staff who screen and enroll the participant but do not conduct baseline, mid-point, or post-treatment assessments. Specifically, once a participant is enrolled, the staff will enter the participant’s ID and pain severity into a randomization program written in R-3.4.3. The program will return the randomization assignment (i.e., allocation). A research staff member, who is not involved in the assessment of outcome, will then inform the acupuncturist via secure email, about the treatment allocation. The acupuncturist will email back, acknowledging receipt of the participant ID and allocation and will then initiate contact with the participant to schedule the first intervention session.

Blinding: both the participant and the research assistants assessing the participant will be masked (blinded) to treatment allocation. We take the following measures to ensure blinding of the participants: (1) the acupuncturists are instructed to hide all their treatment equipment from the participants before treatments; (2) participants are placed in the prone position that prevents them from seeing the intervention process; (3) we have standardized the acupuncturists’ communication with the participants so it is equal between the real and sham arms. To assess the success of these blinding measures, a blinded research assistant will administer a blinding questionnaire at the final post-treatment study visit. To ensure blinding of the research staff that assess the participants, we separate the main assessment database from the treatment database where the allocation is revealed. Additionally, the randomization staff are instructed to avoid communicating any information on treatment assignment to the assessment staff.

### Data collection methods

Plans for assessment and collection of measures: most self-report measures will be collected via electronic surveys (REDCAP™) during study visits, using an encrypted tablet connected to a secure server where the data will reside. The only exception is the weekly assessment of back pain bothersomeness, where a link to the survey will be emailed to participants. The QST data from each participant will be collected by trained research assistants at the baseline (pretreatment), mid-point (between the 8th and 9th treatment session), and post-treatment visit at our facility. All research assistants will undergo extensive training on how to administer online surveys, and more importantly, how to conduct standardized QST tests. They will watch training videos, practice on each other multiple times, then will be signed off by the first author or the manager before conducting tests on participants. Furthermore, the research assistants and the protocol directors hold weekly meetings to address issues related to data collection and standardized QST administration.

#### Strategies to encourage participant retention

We will promote adherence and retention by sending appointment reminder emails, text messages, and phone calls the day before scheduled assessment visits. In these reminders, we communicate the expectation and importance of attending all study assessment visits and not missing more than three treatment sessions. We will review the protocol timeline with each participant at the end of all visits. The team will also hold weekly meetings to troubleshoot emerging issues pertaining to retention. Finally, the participants’ compensation is also contingent on the number of assessment and treatment sessions they complete.

#### Data management

All data will be collected electronically using the HIPPA-compliant REDCAP platform. Participants will receive automated email reminders to fill out their surveys via a secure link. Some surveys and case report forms will be filled out on site, either on a desktop computer or a tablet during study assessment visits. The research team will assess data quality every 6 months by plotting data ranges and checking for missing values on the REDCAP output. These reviews will be done without unblinding. The team will also hold weekly meetings to troubleshoot emerging issues pertaining to data collection and/or participant recruitment and retention issues. The team will also hold weekly meetings to troubleshoot emerging issues pertaining to data collection.

#### Statistical methods

Given that the primary focus is on mechanisms, rather than efficacy, the primary analyses will be carried out using the per-protocol sample, consisting of participants who have completed all three assessment sessions (pre, post and midpoint), and a minimum of 13 out of the 16 treatment visits (i.e., treatment completers). Next we present analysis plan only for the primary hypotheses.

##### Hypothesis 1a

Compared to sham, real treatment will lead to greater reduction in TS from baseline (week 0) to end of week 4.

##### Planned analysis

This hypothesis will be tested using the two-tailed, two-sample *t* test, comparing the change in TS from baseline to week 4 between the real and sham arms.

##### Hypothesis 1b

Reduction in TS from baseline to week 4 will mediate reduction in weekly back pain bothersomeness scores over the course of treatment (from baseline to post-treatment, around week 10).

##### Planned analysis

We will use a McArthur mediation analysis strategy [60] to test this hypothesis. We will first examine if there is a significant treatment effect on the clinical outcome, testing a mixed effect model, with weekly back pain bothersomeness scores as the dependent variable and treatment assignment as the independent variable. The subject-specific intercept will be included to account for within-patient correlation. We will then test a second mixed effects model, with the longitudinally measured back pain bothersomeness scores as the independent variable. Treatment arm, reduction in TS from baseline to week 4, and their interaction will be the independent variables. A significant main effect for change in TS or an interaction between change in TS and treatment arm will be interpreted as evidence of mediation.

##### Hypothesis 2

Participants’ expectations of benefits will predict reduction in back pain bothersomeness scores during the treatment period (week 0 to week 10).

##### Planned analysis

This hypothesis will be tested using a mixed effects regression analysis with weekly measured back pain bothersomeness scores as the dependent variable and expectation, treatment assignment, and their interaction as independent variables.

Hypotheses for aim 3 will be tested using the same analytic plan, replacing primary measures with secondary measures.

#### Monitoring

The Data Safety Monitoring Board (DSMB) consists of three international experts in chronic pain, neuroscience, and complementary and alternative medicine. These members are independent from this clinical trial and are tasked with monitoring participant safety and data integrity and ensuring that the clinical trial is conducted with rigorous adherence to the protocol. The DSMB meets at least once a year, and biannually receives reports from the research team on patient safety, protocol deviations, data completion, and enrollment.

#### Stopping rules and interim analysis

The interventions provided in this study are minimally invasive and carry minimal risk to participants. The most common risks in acupuncture are transient local swelling, bruising, pain, and, less commonly transient nausea and dizziness. The study may be stopped by the monitoring board if significant safety concerns arise. However serious adverse events, such as nerve injury or pneumothorax are rare. We do not plan to perform an interim analysis.

#### Harms and trial conduct auditing

Adverse events (AE) will be monitored in 3 ways: (1) the acupuncturist will document, at each treatment session, any AEs observed during the session or from previous visits; (2) the participant will report any AEs on weekly study surveys; and (3) the research team will systematically query participants about AEs at the mid-treatment and post-treatment assessment visits. All AE’s will be followed up with phone calls by the research team to ensure their resolution.

Trial conduct will be monitored as follows. Acupuncturists’ fidelity will be monitored as described under "Interventions" in the “Methods” section. Checklists of the research procedures and protocol will be used to monitor research protocol deviations (PD). The research assistants will complete case reports that will be reviewed by the project manager on a quarterly basis. Both AEs and PDs will be reported to the DSMB and IRB according to regular reporting schedule. Serious AEs will be reported immediately to the principle directors (JK, RM, SM), DSMB, and IRB.

## Discussion

We have presented an ongoing clinical trial to evaluate the mechanisms of real vs sham EA for the treatment of CLBP. Our primary hypothesis is that the reduction in subjective rating of back pain bothersomeness in response to electroacupuncture will be mediated by reduction in ascending pain facilitation, which is measured by temporal summation. Clinically, if proven true, this hypothesis would allow us to utilize temporal summation as a predictor of clinical response to acupuncture treatments, i.e. those with amplified temporal summation would have increased likelihood to achieve symptom relief with acupuncture. We also hypothesize that the clinical effects of acupuncture will be moderated by the participant’s expectation of treatment benefits, i.e., the higher the expectation, the greater the degree of reduction in back pain bothersomeness. This hypothesis may allow us to potentially increase response to acupuncture treatments by augmenting a patient’s expectation of treatment benefits.

This study utilizes novel mechanistic markers involving an array of QST measures, and examines potential psychological moderators of clinical response to acupuncture, with expectation of benefits as a primary moderator. The study is expected to yield important knowledge on the pain regulatory pathways involved in both real (active) and sham (placebo) EA treatments. The majority of the existing studies on EA in CLBP focus on efficacy alone and are often limited by a heterogeneous sample population (i.e., patients with radicular back pain due to nerve root compression or those with acute and subacute back pain are included along with those with axial CLBP), lack of controls for non-specific effects, or small sample sizes. We designed the current study to minimize these limitations. To the best of our knowledge, this is the largest placebo-controlled study to date to evaluate EA as a treatment for back pain. The QST measures we selected will offer valuable insight into the pain regulatory mechanisms of real and sham EA. The study will also provide insight on psychological measures, such as expectation, catastrophizing and self-efficacy in managing pain, as moderators of treatment response to acupuncture. These measures have been studied extensively in the pain literature but are only beginning to gather attention in the field of acupuncture research.

The primary challenges in the conduct of this trial involve recruitment and the execution of QST. We have and will continue to address these challenges as detailed below. We continually assess the success of our recruitment effort, modifying and augmenting methods to optimize recruitment. To date the most effective recruitment method has been the use of social media marketing campaigns (Facebook, Twitter, Instagram, Reddit). The challenge with conducting QST stems from large, between-individual variation in understanding instructions and in the ability to tolerate various test stimuli. We have implemented several tactics to reduce this variability. All research assistants undergo extensive video and hands-on training to ensure standardized administration of the QST instructions and procedures. Participants are given adequate time to practice and adjust to the QST stimuli. The most technically challenging QST protocol is thermal TS. To meet the challenge, we individually adjust the thermal stimuli in our protocol to minimize floor or ceiling effects. We also wait approximately 3–5 min between trials to allow adequate time for the participant’s skin temperature to return to baseline.

## Trial status

Participant recruitment for this study began on 17 March 2015, and is expected to conclude by 31 December 2018. The initial protocol and subsequent revisions follow the guidelines from the funding agency, the National Center for Complementary and Integrative Health (NCCIH), and have been approved by the Stanford Institutional Review Board (IRB). The most recent protocol version of this study is 2.0, approved by the National Center of Complementary and Integrative Health (NCCIH) and Stanford IRB on 3 January 2016.

## Additional file

Additional file 1: SPIRIT Checklist (required for study protocols) - version 2013. (DOCX 21 kb)

## Abbreviations

AE: Adverse event
CLBP: Chronic low back pain
CPM: Conditioned pain modulation
EA: Electroacupuncture
HIPPA: Health Insurance Portability and Accountability Act
IRB: Institutional review board
MINI: Mini International Neuropsychiatric Interview
NIH: National Institutes of Health
PROMIS: Patient Reported Outcome Measures Information System
QST: Quantitative sensory testing
STRICTA: Standards for reporting interventions in clinical trials of acupuncture
TS: Temporal summation
VAS: Visual analog scale
